# Metabolic Flexibility and Exercise Performance in Adults with Gilbert’s Syndrome–Associated Hyperbilirubinemia

**DOI:** 10.1186/s40798-026-01011-2

**Published:** 2026-04-15

**Authors:** Tamara Christina Stelzer, Anna Maria Kripp, Agnes Draxler, Lina Maqboul, Katharina Tatjana Pfeiffer, Andrew Cameron Bulmer, Daniel König, Karl-Heinz Wagner

**Affiliations:** 1 Department of Nutritional Sciences, University of Vienna, Josef-Holaubek Platz 2, 1090 Vienna, Austria; 2 Vienna Doctoral School for Pharmaceutical, Nutritional and Sport Sciences (PhaNuSpo), University of Vienna, Vienna, Austria; 3 Department of Health Sciences, FH Campus Wien, University of Applied Sciences, Vienna, Austria; 4 School of Pharmacy and Medical Sciences, Griffith University, Gold Coast, Australia; 5 Department of Sport Science, Centre for Sport Science and University Sports, University of Vienna, Vienna, Austria

**Keywords:** Bilirubin, Gilbert’s syndrome, Maximal fat oxidation, Performance, Exercise metabolism, Aging

## Abstract

**Background:**

Recent evidence suggests that moderately elevated bilirubin plasma concentrations possess protective effects against non-communicable diseases. One possible explanation for this might be that Gilbert’s Syndrome (GS), a mildly hyperbilirubinaemic condition, leads to an enhanced lipid metabolism. Furthermore, there are first hints that individuals with GS may have a greater performance capacity. We hypothesize that GS participants have a greater maximal fat oxidation and performance capacity.

**Methods:**

To test this, we conducted an age- and gender-matched human case-control study. We included 40 people with GS and 40 controls, aged 18–65 years, who were all moderately physically active. 50% of the participants were over the age of 35. Participants performed a FatMax test on a bicycle ergometer. The study was performed from March 2023 to December 2023 at the University of Vienna.

**Results:**

The group of GS participants over the age of 35 had a significantly higher FatMax (GS: median = 1.03 [maximum = 0.44; minimum = 2.42] W/kg body weight; C: median = 0.48 [minimum = 0.33; maximum = 1.61] W/kg body weight, *p* = 0.002) and a significantly greater oxygen consumption (GS: mean = 30.2 ± standard diviation (sd) = 8.09 ml/min/ kg body weight; C: mean = 23.4 ± sd = 5.91 ml/min/kg body weight, *p* = 0.005) at the respiratory compensation point.

**Conclusion:**

This is the first study to demonstrate that older GS individuals can generate more power whilst harnessing fatty acid metabolism and this may enhance their performance over prolonged periods of sub-maximal exercise.

**Supplementary Information:**

The online version contains supplementary material available at 10.1186/s40798-026-01011-2.

## Key Points

- This is the first study, that investigates the effect of mildly elevated bilirubin concentrations on exercise performance and metabolic flexibility in terms of maximal fat oxidation (MFO) during exercise.
- Previous studies have already shown that the potential protective effect of mild hyperbilirubinemia is more pronounced in adults over the age of 35. We observed this more pronounced effect also for exercise performance and metabolic flexibility.
- The study clearly supports previous research that already found a greater fat oxidation in participants with Gilbert’s syndrome.

**Supplementary Information:**

The online version contains supplementary material available at 10.1186/s40798-026-01011-2.

## Introduction

Traditionally classified as a potentially neurotoxic end-product of heme degradation, bilirubin has more recently emerged as a molecule of interest due to its putative cytoprotective, antioxidant, and anti-inflammatory properties — especially in the context of moderate hyperbilirubinemia observed in Gilbert’s syndrome (GS) [1–4]. GS is an inherited benign condition of non-hemolytic unconjugated hyperbilirubinemia, caused by a genetic variation in the *UGT1A1* gene which in turn leads to a reduced activity of the hepatic enzyme UDP-glucuronosyltransferase 1A1 (*UGT1A1*) due to a promoter region mutation [5]. Mild hyperbilirubinemia and its associated protective effects are associated with mutations in the *UGT1A1* gene [6]. The most common genotype observed in GS is the (TA) 6 > 7 insertion in the promoter region of the *UGT1A1* gene (UGT1A1*28 polymorphism) [7]. This polymorphism reduces *UGT1A1* enzymatic activity and impairs hepatic conjugation of bilirubin, resulting in an accumulation of unconjugated bilirubin (UCB) in the circulation [8]. The global prevalence of the UGT1A1*28 polymorphism is very high, ranging from 5 to 20% of the population, depending on the ethnicity [5, 9]. Physiologically, serum bilirubin concentrations range up to 20.4 µmol/L (1.2 mg/dl) while normal unconjugated bilirubin (UCB) ranges from 5 to 17 µmol/L (0.2-1.0 mg/dL). Bilirubin concentrations below 10 µmol/L (0.6 mg/dL) have been associated with a greater risk of cardiovascular events and metabolic disorders [10]. This association is even more pronounced for concentrations below 7 µmol/l (0.4 mg/dl) [5, 11].

Numerous studies have demonstrated that moderately elevated bilirubin concentrations — and the associated metabolic profile — are linked to a reduced risk of various chronic diseases, such as cardiovascular diseases (CVD), type 2 diabetes mellitus (T2DM), certain forms of cancers, and the metabolic syndrome [12–16]. Several studies, including those conducted by our group and others, have shown that GS individuals tend to exhibit reduced BMI and a greater fat-free mass compared to matched healthy controls [17–20]. These body composition characteristics may partly explain the reduced risk of chronic diseases observed in GS populations. Another contributor to the reduced prevalence of chronic diseases in individuals with GS is the potent antioxidant capacity of bilirubin [19]. From a metabolic perspective bilirubin may also enhance lipid catabolism. Hana et al. demonstrated that acetyl carnitine, 3-hydroxybutyric acid, aceto-acetic acid, and acetone were significantly higher in GS subjects compared to age- and sex-matched controls [21]. Therefore, GS individuals have a specific metabolic profile, where downstream metabolites associated with fatty acid oxidation and ketogenesis are significantly elevated [21]. Furthermore, various groups showed consistently that triglycerides, total and LDL-cholesterol, as well as subfractions of LDL, of both natures, pro- and low-atherogenic, were significantly lower in people with elevated bilirubin concentrations [22–24]. Therefore, it is plausible that impaired bilirubin metabolism alters lipid metabolism and plays a crucial role in protecting humans with GS from CVD. Moreover, several studies [4, 18, 22, 41] have shown that the effect of bilirubin on metabolism and lipid profile is even more pronounced in older adults. Therefore, it seems that there is an “age effect” of bilirubin.

### Bilirubin and Exercise

Regular physical activity is associated with increased serum bilirubin concentrations. Moreover, elevated serum bilirubin concentrations are associated with improved exercise performance [25]. In a Czech study, serum bilirubin concentrations were significantly greater in elite athletes compared to normal controls. Furthermore, Woronyczová et al. reported that the prevalence of GS was higher in elite athletes [26]. Exercise directly upregulates heme oxygenase-1 (HO-1) expression, thereby promoting the breakdown of heme and contributing to increased bilirubin concentrations [27]. Additionally, the mechanical stress induced by physical activity may enhance erythrocyte turnover and heme release, further stimulating bilirubin production [28, 29].

The main enzymes regulating plasma bilirubin concentrations, such as HO-1, biliverdin reductase (BVRA) and *UGT1A1* may also be modulated by exercise [28]. In a rodent study, Hinds et al. compared high and low capacity running rats. They found that BVRA activity is significantly enhanced, while UGT1A1 activity was suppressed in the high capacity running rats resulting in higher serum bilirubin concentrations [25]. These findings suggest that a higher aerobic capacity enhances BVRA and reduces *UGT1A1*. In a human study, Swift et al. observed significantly increased bilirubin concentrations after a 6-month exercise intervention in overweight and obese postmenopausal women [29]. Notably, the increase in bilirubin was more pronounced in insulin-resistant individuals than in insulin-sensitive participants [29].

Bilirubin, as a strong antioxidant, also responds to reactive oxygen species (ROS) produced during exercise and may contribute to the protection of muscle fibers from oxidative damage [25]. Furthermore, it is now well established, that bilirubin acts as a signaling molecule by binding to peroxisome proliferator-activated receptor α (PPARα) and enhancing its transcriptional activity [30]. This is particularly relevant since PPARα increases hepatic glycogen content which may, in turn, contribute to improved aerobic capacity [25]. However, this only becomes relevant at exercise durations of 90 min and more [31]. Upon activation of PPARα, through bilirubin or other stimuli, FA uptake into cells [32], mitochondrial β-oxidation [33], and ketogenesis [34] are improved. Therefore, bilirubin binding to PPARα may lead to enhanced fat metabolism which supports the hypothesis that GS individuals have a different energy and fat metabolism compared to age and gender matched controls.

As regular exercise has metabolic advantages on fatty acid (FA) oxidation and bilirubin is also associated with an enhanced FA metabolism, we hypothesize that individuals with GS exhibit a distinct metabolic phenotype characterized by enhanced fatty acid oxidation during exercise and therefore a greater metabolic flexibility.

## Materials and Methods

### Study Design and Participants

This metabolic flexibility study was part of the larger Austrian Science Fund-funded “BiliMetHealth” project. This was an age and gender matched case-control study conducted at the Nutrition and Training Laboratory (NuTraLab) of the University of Vienna. From March 2023 to December 2023, all participants were recruited through posts on social media and the homepage of the University. The posts on the homepage and social media initially sought to address primarily individuals with moderately elevated bilirubin concentrations. After having recruited a person with GS, an age- and gender matched control person was searched. Eligibility for the study was assessed during a preliminary examination that included a medical history interview and a routine blood test including total blood count, white blood cell count, liver function test, blood glucose, urea, cholesterol, triglycerides, albumin, and C-reactive protein (CRP). All analyses were conducted by a certified laboratory (SYNLAB, Vienna, Austria).

**Fig. 1:**
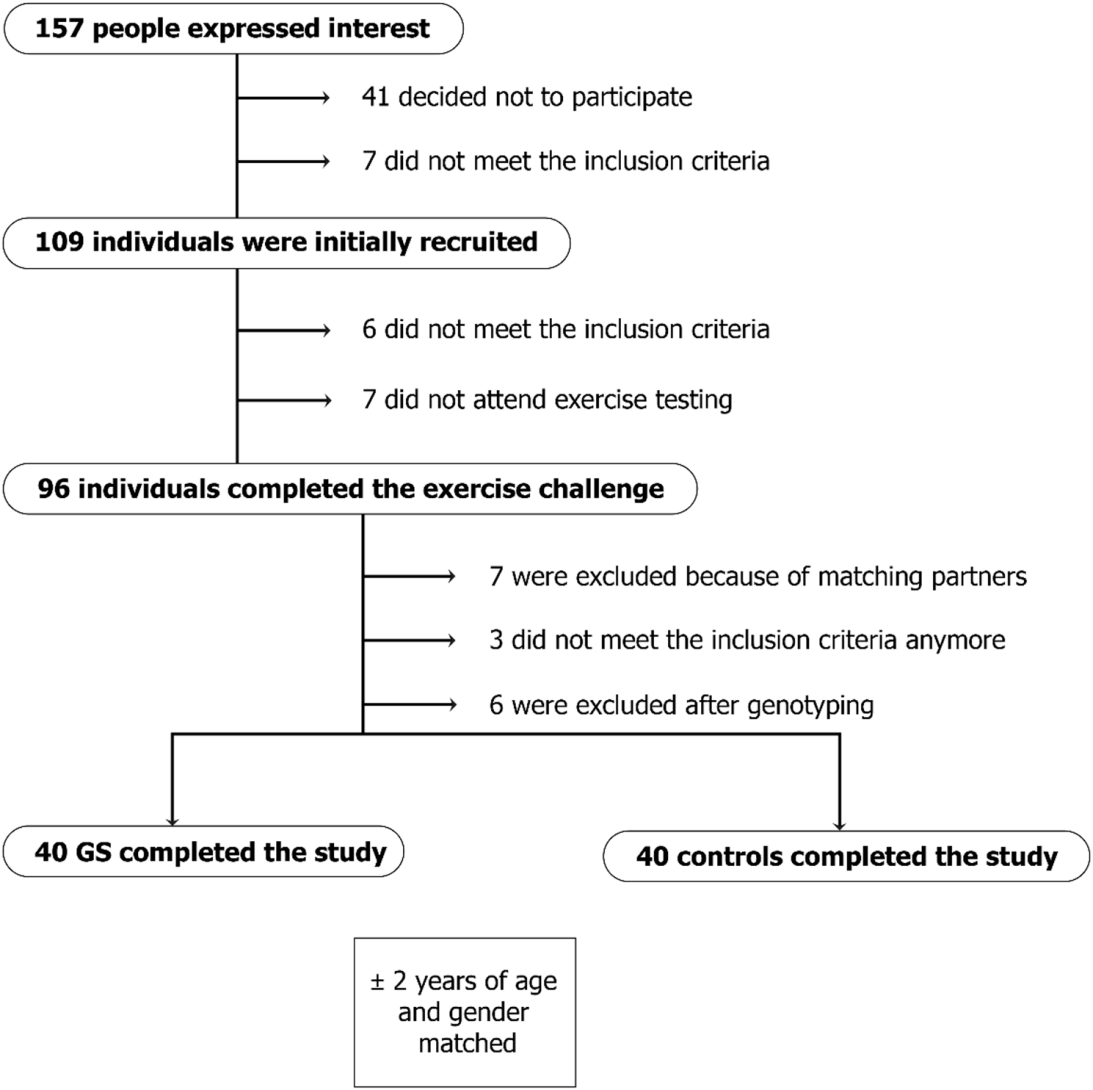
The Flow chart shows how many people were recruited initially, the dropouts and how many were finally included in the study

In addition, UCB concentrations were assessed using high-performance liquid chromatography (HPLC). Individuals were classified as having GS if their UCB concentrations exceeded 17.1 µmol/L and total serum bilirubin surpassed the clinical diagnostic threshold of 1.2 mg/dL. Additionally, genotyping for UGT1A1*28 was done, however this was not part of the inclusion criteria, as it is also stated in a recently published paper by Vitek and Tiribelli 2023, that genetic testing is not part of the GS diagnosis [35]. Furthermore, liver disease was excluded by assessing the concentrations of aminotransferase (AST), alanine transaminase (ALT), gamma-glutamyl transferase (GGT) and lactate dehydrogenase (LDH). All participants were healthy, without any acute or chronic (metabolic or inflammatory) diseases, and between 18 and 65 years of age. They were moderately physically active (2–3 times per week) and non-smokers. The study was approved by the Ethics commission of the University of Vienna (#00905) and was conducted with approved guidelines by the Declaration of Helsinki. All participants gave written informed consent. Recruitment process and number of participants are presented in Fig. 1.

### Exercise and FatMax Testing

As a second and last study visit, after the preliminary examination, a metabolic flexibility challenge was conducted using a FatMax test on a bicycle ergometer following a 12-hours overnight fast. The FatMax test consisted of a graded step protocol for non-athletes recently published by Chrzanowski-Smith et al. [36]. Before the exercise challenge, participants had to complete the international physical activity questionnaire (IPAQ) [37] which then determined the initial power for the graded test. During the test, breath-by-breath gas exchange (Cortex MetaLyzer 3B, Leipzig, Germany) and heart rate (Bluetooth Smart HF-sensor H10, Polar Electro Austria GmbH, Vienna, Austria) were continuously measured. Before the cycling test, venous blood samples were taken. Substrate utilization was calculated with stoichiometric formula [38] from average $\:\mathrm{V}$O_2_ values and the respiratory exchange ratio (RER) 4-min in each step. Fat oxidation was calculated for every stage of the graded exercise test. Maximal fat oxidation (MFO) was defined as the highest fat oxidation calculated from the stochiometric formula and FatMax, the corresponding intensity, was assessed by plotting MFO against power output (W) on a graph and determining the highest peak. The aerobic threshold (AT) was determined as the first increase in ventilation and the respiratory compensation point (RCP) as the second increase in ventilation, respectively. Since the test was terminated at a RER of 1.00, maximal oxygen uptake ($\:\dot{\mathrm{V}}$O2max) could not be measured. However, it was estimated as previously proposed by Nunes et al. [39]. The testing conditions were always similar. All subjects had to fast 12 h prior to the test and had to follow specific dietary guidelines the day before the test. They were asked to eat only moderate amounts of carbohydrates and additionally they should not exceed their habitual amount of caffeine. Moreover, they were asked to avoid alcohol the day before the testing day. Whether they complied with this was checked using a 24-h-recall. In addition, they were asked not to engage in strenuous physical activity the day before. On the testing day, they were asked to come to the study center fasted (12 h).

### *UGT1A1* Genotyping

After blood sampling, whole blood coated in EDTA was immediately frozen at -20 °C and stored for one year at − 80 °C. For analysis, samples were thawed and DNA was isolated with the QIAmp blood mini kit (QIAGEN, Venlo, Netherlands) according to the protocol of the kit. After isolation, the quality and quantity of the DNA were checked with Nanodrop© (Thermofisher, Waltham, USA). Nanodrop calculated DNA absorption at 260 nm. DNA purity was assessed using the 260/280 ratio. The optimal ratio for DNA purity is 1.80 [40]. Values between 1.60 and 1.95 were accepted. Genotyping was performed with Pyromark Q24 and the therascreen *UGT1A1* kit (QIAGEN, Venlo, Netherlands). Samples were screened for the UGT1A1*28 TA6 > TA7 variant.

### Anthropometric Parameters

For anthropometric measurements the International Standards for Anthropometric Assessments [41] were followed. Participants were barefoot, wearing light indoor clothing, after an overnight fast (12 h) and without alcohol intake for the previous 24 h. Height was measured to the nearest 0.1 cm with a stadiometer (Seca, freestanding stadiometer with digital display, 274, Hamburg, Germany), while body weight was measured to the nearest 0.1 kg (Seca, mBCA 515, Hamburg, Germany). Body mass index (BMI) was determined as body mass divided by the body height squared (expressed in kg/m^2^). Body composition was assessed using bioelectric impedance analyses (BIA) (Seca, mBCA 515, Hamburg, Germany). Hip and waist circumference were measured with a tape, and the waist-to-hip ratio (WHR) was calculated.

### Blood Analysis

Blood samples were either immediately analyzed at the certified laboratory (SYNLAB, Vienna, Austria) or centrifuged and as plasma or serum aliquoted into cryovials and finally frozen and stored at – 80 °C for later analysis. Analysis of cholesterol, triglycerides, and glucose before and after the exercise test, as well as at baseline, were done by SYNLAB, Vienna, Austria. UCB concentrations, before and after the exercise test, were measured in our lab by HPLC as already described previously by Wallner et al. and Schoissengeier et al. [42, 43]. Serum was used for the analyses. 50 µl serum were mixed with 200 µl mobile phase (consisting of 93.8% methanol, 2.3% N-Dictylamine, 0.6% Acetic acid and 3.4% distilled water) and centrifuged at 14,000 rpm for 10 min. Thereafter, 120 µl supernatant was pipetted into vials and used for analysis. Serum UCB concentrations were measured by HPLC (Shimadzu Nexera HPLC/UHPLC, Vienna, Austria) at 450 nm, equipped with a Fortis C18 HPLC column (4.6 × 150 mm, 3 mm), and a Phenomenex Security Guard cartridge for C18 HPLC columns (4 × 3 mm), and a photodiode array detector (PDA, Shimadzu). Bilirubin standards were prepared using a 98% bilirubin powder (Sigma Aldrich, Darmstadt, Germany). The concentration was expressed in µmol/L.

### Measurement of Resting Metabolic Rate

Resting metabolic rate (RMR) was measured using the Q-NRG indirect calorimeter with a Canopy mask (Cosmed, Rome, Italy). Participants were in a supine position for 5 min before the measurement and during the whole measurement, which lasted 10 min. The flow rate was adjusted for each participant, in order to meet a fraction rate of expired carbon dioxide of no more than 1.0%, as recommended by the manufacturer. Oxygen consumption ($\:\mathrm{V}$O_2_) and carbon dioxide production ($\:\mathrm{V}$CO_2_) were recorded breath-by-breath by the device. RMR was calculated in the Omnia software over 5 min of steady state with the aid of $\:\mathrm{V}$O_2_ and $\:\mathrm{V}$CO_2_. Respiratory quotient (RQ) was also determined over the chosen 5 min interval.

### Lifestyle Parameters

Physical activity was assessed using the international physical activity questionnaire (IPAQ) [37]. Furthermore, participants were asked to complete a 24-hour-recall questionnaire to assess whether they followed the dietary instructions for the day before the test and to assess their carbohydrate intake on the day before. In addition, participants had to complete the quality-of-life questionnaire from the World Health Organization (WHO) [44] and a food frequency questionnaire (FFQ).

### Statistics

Statistical analysis was performed using R (Version 2025.05.0 + 496). Results are presented as mean ± standard deviation, when normally distributed or as median [minimum; maximum], when not. P-values ≤ 0.05 were considered statistically significant. Distribution was tested using the Shapiro-Wilk test and visually by analyzing the histograms. For baseline data, an unpaired T-test was conducted in case of normal distribution, and a Mann-Whitney-U-test was used in other cases. A generalized linear model was used to assess differences between the two groups (GS and C) and to investigate the effect of age group / age on the outcome variables and their interaction (group × age). Furthermore, all variables of interest (MFO, FatMax, AT, and RCP) were correlated with UCB in a Spearman rank correlation test.

## Results

**Table 1:** Baseline metabolic parameters of the GS and the C group. Values are displayed in mean (standard deviation) if the data was normally distributed and in median [min; max] if not

	GS (*n* = 40)	C (*n* = 40)	*p*-value
Age (years)	36.4±12.7	37 ± 12.6	0.832
% female	42.5	42.5	
Body height (m)	1.76 ± 0.09	1.75 ± 0.10	0.746
Body weight (kg)	71.7 ± 12.9	75.6 ± 15.2	0.223
Total bilirubin (mg/dl)	1.97 ± 0.78	0.72 ± 0.16	< 0.001*
UCB (µmol/L)	31.6 ± 16.0	9.91 ± 3.47	< 0.001*
BMI (kg/m^2^)	23.0 ± 2.79	24.5 ± 3.75	0.050*
FM (%)	24.2 ± 7.37	25.9 ± 9.35	0.359
FFM (%)	75.8 ± 7.37	73.3 ± 11.3	0.247
Waist circumference (cm)	81.7 ± 11.7	83.8 ± 12.7	0.449
Hip circumference (cm)	102 ± 7.57	103 ± 8.64	0.538
WHR	0.80 ± 0.06	0.81 ± 0.07	0.444
Systolic BP (mmHg)	114 ± 12.2	113 ± 11.8	0.794
Diastolic BP (mmHg)	73.8 ± 9.70	74.3 ± 10.8	0.820
Glucose (mmol/L)	4.51 ± 0.595	4.48 ± 0.42	0.817
Triglycerides (mmol/L)	0.71 [0.38; 4.05]	0.84 [0.37; 2.45]	0.055
Total chol. (mmol/L)	4.16 ± 0.82	4.45 ± 0.73	0.099
LDL chol. (mmol/L)	2.24 ± 0.52	2.49 ± 0.54	0.041*
HDL chol. (mmol/L)	1.58 ± 0.33	1.49 ± 0.26	0.164
VO2max pred Ƚ (ml/min/kg)	39.8 ± 8.87	36.5 ± 9.65	0.121
RMR (kcal/d)	1477 ± 382	1817 ± 371	0.397
RQ	0.77 ± 0.06	0.78 ± 0.07	0.306
IPAQ	2.00 [1.00;3.00]	2.00 [1.00;3.00]	0.133

A T-test was applied when normal distribution was true, and a Mann-Whitney-U-Test was performed. Ƚ VO2max was calculated according to a formula by Nunes et al. [39]. Significance was marked in bold letters with a *. *UCB* (unconjugated bilirubin), *BMI* (body mass index), *FM* (fat mass), *FFM* (fat free mass), *WHR* (waist-to-hip-ratio), *BP* (blood pressure), *chol.* (cholesterol), *LDL* (low density lipoprotein), *HLD* (high density lipoprotein), *VO2max pred* (maximal oxygen uptake predicted), *RMR* (resting metabolic rate), *RQ* (respiratory quotient), *IPAQ* (international physical activity questionnaire)

Table 1 shows the baseline parameters of the GS and the control (C) group. BMI was significantly higher in C participants compared to GS (*p* = 0.050). Furthermore, body composition and blood pressure were not different between groups. Regarding lipid profile, LDL-cholesterol concentrations were significantly higher in the C group (2.24 ± 0.52), than in the GS group (2.49 ± 0.54; *p* = 0.041). The remaining blood lipid values were not significantly different between the groups. However, there is a trend towards lower total cholesterol and triglyceride (TG) concentrations and higher HDL cholesterol (HDL-C) in the GS compared to the C group.

No differences in the IPAQ score, predicted $\:\mathrm{V}$O2max, RMR and RQ were observed between the groups.

Results from the FFQ revealed no significant differences between groups in any of the analyzed food groups. Furthermore, WHO lifestyle questionnaire demonstrated no significant differences between groups in their lifestyle. Detailed information is added to the supplementary part.

**Table 2:** Results of the genotyping displayed in % per gene variant

	Homozygote (TA/TA)	Heterozygote (-/TA7)	Wildtype (-/-)	p-value
GS (*n* = 40)	82.5%	17.5%	0.00%	< 0.001*
C (n=40)	5.00%	47.5%	47.5%	

Table 2 shows that there is a significant difference between GS and C group in genotype of the UGT1A1*28 variant.

**Fig. 2:**
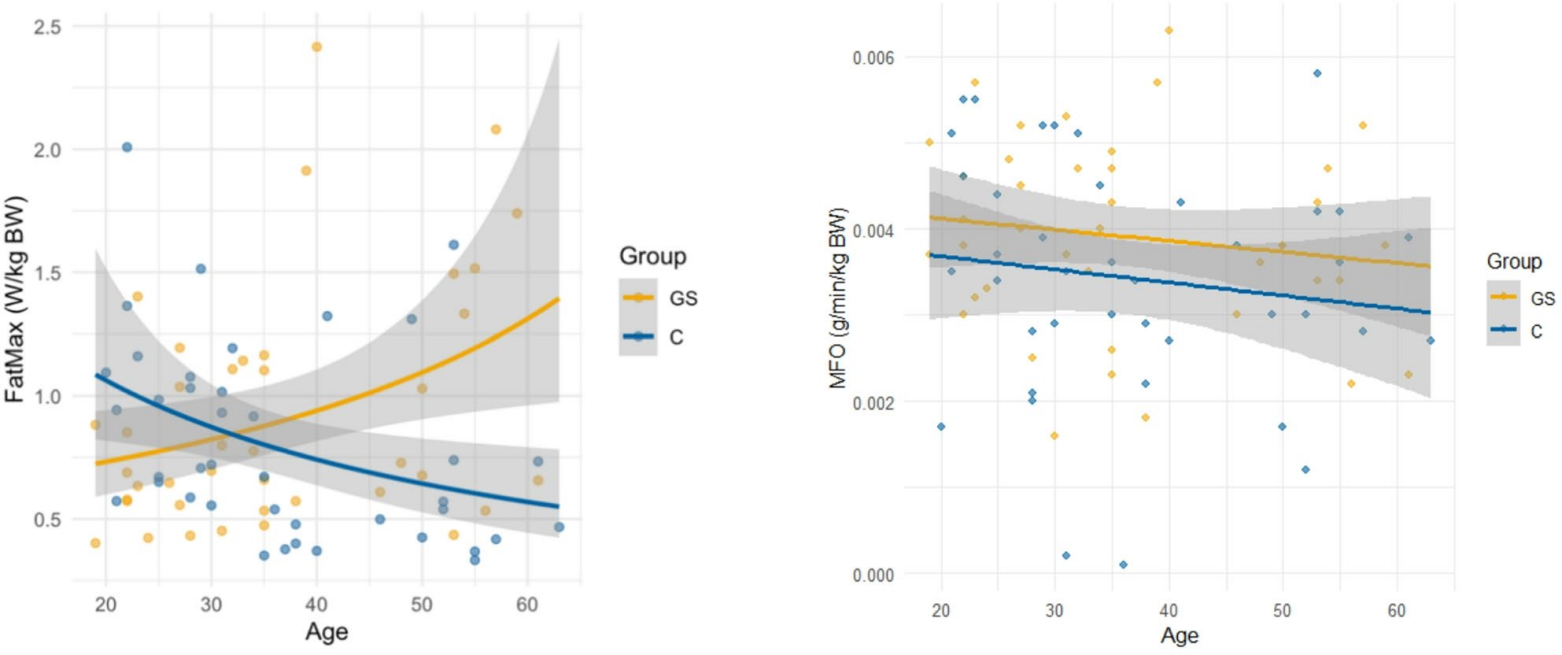
FatMax (W/kg BW) and MFO (g/min/kg BW) are plotted against age

Figure 2 shows that FatMax increases with age in the GS group, whereas it declines with age in the C group. This effect is significant in a general linear model where group (GS or C) and age group (< 35; >=35) were included as factors. The Tukey HSD post hoc test revealed a significant difference in FatMax between the GS and the C group in the older subgroup (*p* = 0.003). Furthermore, the difference between the C group over the age of 35 and the younger C group was significant (*p* = 0.032), whereas age group differences in the GS group were not significant (*p* = 0.055). Although MFO appeared lower at older ages in both groups, this association was not statistically significant (*p* = 0.572). However, as Fig. 2b shows, MFO in the GS group is slightly higher than in the C group. By comparing MFO of GS to C, a slight trend towards a higher fat oxidation in the GS group was observed (0.0039 ± 0.0011 vs. 0.0034 ± 0.0014; *p* = 0.086). Furthermore, MFO is significantly correlated with circulating UCB concentrations (*r* = 0.228; *p* = 0.043).

**Fig. 3:**
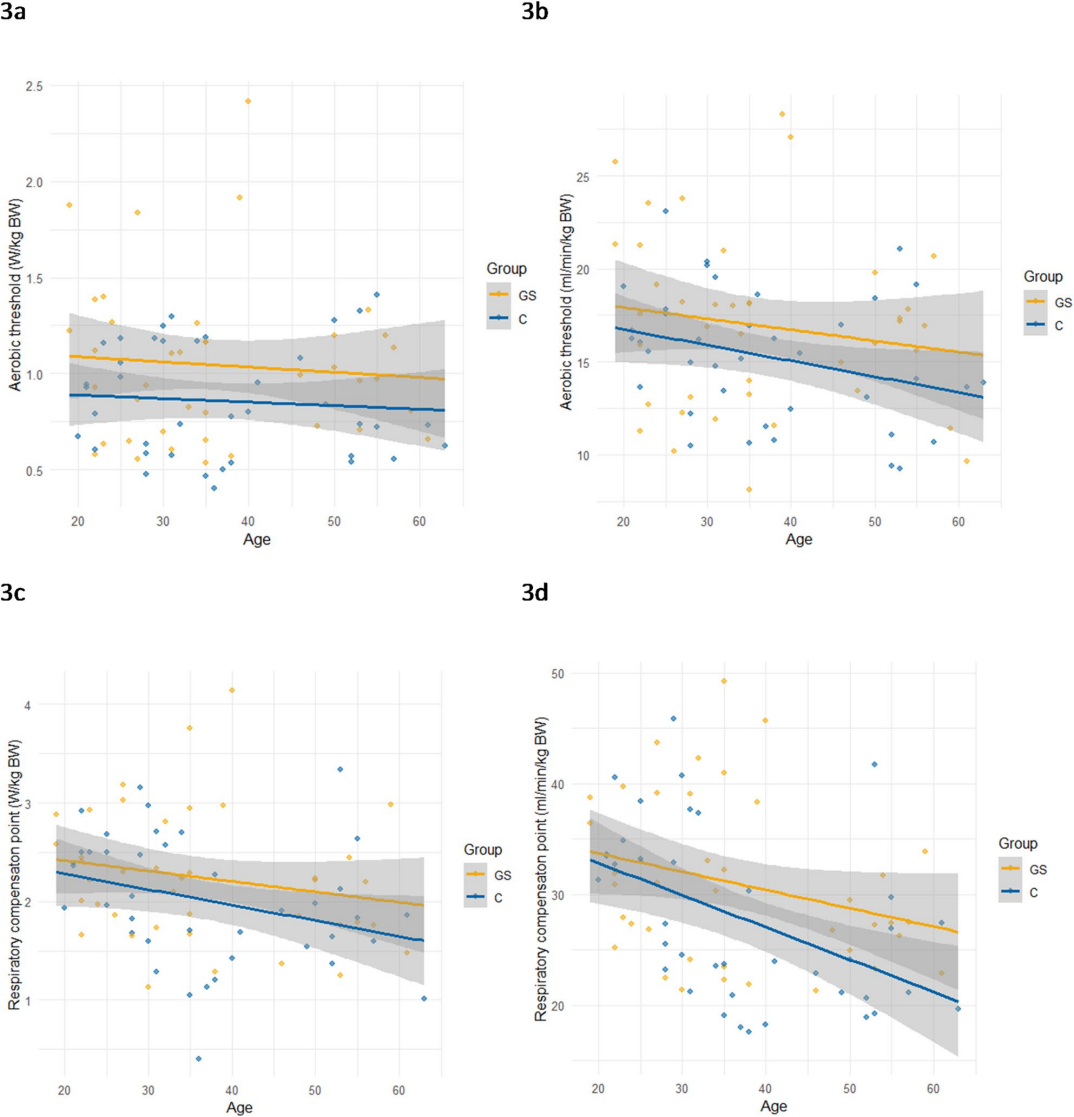
Aerobic threshold (3a in W/kg, 3b in ml O_2_/min and kg BW) and respiratory compensation point (3c in W/kg, 3d in ml O_2_/min and kg BW) are plotted against age

No differences between groups were observed at the AT in W/kg BW (Fig. 3a) as well as in ml/min/kg BW (Fig. 3b). Regarding RCP in W/kg BW (Fig. 3c) no group differences were observed, whereas in ml/min/kg BW there was a significant difference between the younger (32.0 ± 6.97) and the older C group (23.4 ± 5.91; *p* < 0.001) and within the older subgroup between GS (30.2 ± 8.09) and C (23.4 ± 5.91; *p* = 0.005). The latter is shown in Fig. 3d.

## Discussion

The main finding of the present study was that older adults with GS had a higher FatMax and exhibited higher fat oxidation rates, even though the latter were not significant, during submaximal exercise compared to age-matched controls, suggesting an enhanced metabolic flexibility potentially mediated by elevated bilirubin concentrations.

Hana et al. revealed that GS individuals demonstrate enhanced lipid catabolism, via elevated circulating ketone bodies [21]. This may help to explain the better overall lipid profile and provides a basis for hypothesizing that individuals with GS could exhibit higher fat oxidation rates at rest and during exercise. However, no significant differences in RQ or MFO were observed. MFO was higher in GS participants, however, did not reach statistical significance. As MFO is dependent on many different parameters, such as age, sex, hormonal status in females, and training level, it might be possible that our study group was too heterogenous to achieve significance. Dividing the sample into further subgroups such by parameters such as gender or physical activity level, would have led to a smaller sample size that would have drastically reduced statistical power. However, a significantly greater FatMax was measured in older GS participants (> 35 years old), suggesting that the effect of bilirubin on metabolism and exercise performance becomes more pronounced with age. This indicates that people with GS over the age of 35 can oxidize fat at higher intensities compared to people in the C group in this age class. This is in accordance with literature, where bilirubin is sometimes described as a “fat burning hormone” [45, 46], as the primary receptor targeted by bilirubin is PPARα [46]. PPARα is known to promote fat oxidation [47].

Similarly, performance at the AT measured in W/kg BW and in ml/min/kg BW decreased with age, and this decrease was greater in the C group than in the GS group. Kong et al. stated that improvements in energy metabolism, antioxidant capacity and anti-inflammatory processes, among others, enhance aerobic endurance [48]. Since it has been shown that people with GS have a different energy metabolism, towards an enhanced energy and lipid metabolism [17], a better antioxidant status [19, 49, 50], and ameliorated anti-inflammatory processes [51, 52], this might explain their slightly better performance at the aerobic threshold.

Furthermore, at RCP oxygen uptake was significantly higher in GS participants over the age of 35 compared to the C group. These findings indicate the more pronounced effect of bilirubin metabolism in older adults. This “age effect” has repeatedly been described in literature [42]. With increasing age, the protective effect of bilirubin metabolism may become more pronounced. At the same time with increasing age, the risk of developing non-communicable diseases also increases [53]. Therefore, the putative protective role of bilirubin and its metabolism likely gain increasing relevance with advancing age. Bilirubin may thus be regarded as an endogenous protective biomolecule contributing to resilience against frailty and functional decline in activities of daily living [54]. Notably, Ling et al. suggested that only total serum bilirubin concentrations within the physiological range may confer protection against frailty [55].

As in previous papers [43, 56, 57], the present study again showed a better lipid profile. Furthermore, a significantly reduced BMI was present in GS participants compared to controls. Therefore, individuals with GS possess a specific phenotype which is characterized by a lower BMI and improved body composition. In addition to BMI, a favorable lipid profile, also accounts for the specific phenotype of GS participants, as observed by several authors in the last decades [4, 21, 43, 57, 58]. Consistent with previous observations, the study population displayed a favorable lipid profile, with significantly lower LDL cholesterol, trends toward higher HDL, and lower triglyceride and total cholesterol concentrations.

## Strengths and Limitations

Since we were the first to perform a FatMax test in context with GS participants the design of our study was novel. Furthermore, with the genotyping there was a second tool with which we could confirm GS in addition to serum bilirubin concentrations. An amelioration for the study might have been to measure VO2max before the FatMax test and consider VO2max as the parameter to define initial power rather than physical activity level.

## Conclusion

This is the first study to test GS compared to C on the capacity to oxidize fat during exercise. These results show a tendency towards greater fat oxidation and FatMax. Particularly, lipid metabolism during exercise was enhanced in the middle/older age cohort, supporting previous research indicating a physiologically important effect of bilirubin during aging that may preserve functional capacity. In addition, also the greater performance at RCP underlines this “age effect” of bilirubin metabolism.

## Supplementary Information

Below is the link to the electronic supplementary material.

Supplementary Material 1.

## Data Availability

The datasets used and/or analysed during the current study are available from the corresponding author on reasonable request.

## Abbreviations

ALT: Alanine transaminase
AST: Aminotransferase
AT: Aerobic threshold
BIA: Bioelectric impendency analysis
BMI: Body mass index
BVRA: Biliverdin reductase
C: Control group
CRP: C-reactive protein
CVD: Cardiovascular diseases
FA: Fatty acids
FFQ: Food frequency questionnaire
GGT: Gamma-glutamyl transferase
GS: Gilbert’s Syndrome
HO-1: Heme oxygenase 1
HPLC: High-performance liquid chromatography
IPAQ: International physical activity questionnaire
LDH: Lactate dehydrogenase
MFO: Maximal fat oxidation
NuTraLab: Nutrition and Training Laboratory
RCP: Respiratory compensation point
RER: Respiratory exchange ratio
RMR: Resting metabolic rate
ROS: Reactive oxygen species
RQ: Respiratory quotient
T2DM: Type 2 diabetes mellitus
UCB: Unconjugated bilirubin
UGT1A1: Uridine diphosphate glucuronosyl tranferase 1A1
VCO2: Carbon dioxide production
VO2: Oxygen consumption
VO2max: Maximal oxygen uptake
WHO: World health organization
WHR: Waist to hip ratio
