# Supplementary material for: Metabolic Flexibility and Exercise Performance in Adults with Gilbert’s Syndrome–Associated Hyperbilirubinemia
Source: Sports Med Open. 2026 Apr 15;12:43. doi: 10.1186/s40798-026-01011-2 (PMC13083743; doi:10.1186/s40798-026-01011-2)
Supplement: Supplementary file 1 — Supplementary Material 1. [file 40798_2026_1011_MOESM1_ESM.pdf]

Metabolic Flexibility and Exercise Performance in Adults with Gilbert's Syndrome – Associated Hyperbilirubinemia

Tamara Christina Stelzer[1,2], Anna Maria Kripp[1,5], Agnes Draxler[1,3], Lina Maqboul[1], Katharina Tatjana Pfeiffer[1], Andrew Cameron Bulmer[4], Daniel König[1,5], Karl-Heinz Wagner[1]

[1] Department of Nutritional Sciences, University of Vienna, Vienna, Austria

[2] Vienna Doctoral School for Pharmaceutical, Nutritional and Sport Sciences (PhaNuSpo), University of Vienna, Vienna, Austria

[3] Department of Health Sciences, FH Campus Wien, University of Applied Sciences, Vienna, Austria

[4] School of Pharmacy and Medical Sciences, Griffith University, Gold Coast, Australia

[5] Department of Sport Science, Centre for Sport Science and University Sports, University of Vienna, Vienna, Austria.

Correspondence: Dr. Karl-Heinz Wagner, Department of Nutritional Sciences
University of Vienna, Josef-Holaubek Platz 2, 1090 Vienna, Austria (e-mail: karl-heinz.wagner@univie.ac.at, phone: +43-1-4277-54930)

Supplementary tables

	GS ≥35	C ≥ 35	p-value	GS < 35	C < 35	p-value
**Age (years)**	47.3 ± 9.11	47.1 ± 9.27	0.930	26.5 ± 4.91	27.9 ± 6.86	0.458
**Gender (%female)**	36.84	36.84		47.62	42.86	
**Body height (m)**	1.77 ± 0.12	1.76 ± 0.12	0.946	1.75 ± 0.08	1.75 ± 0.08	1.000
**Body weight (kg)**	76.2 ± 14.9	78.4 ± 12.9	0.965	67.6 ± 9.48	73.0 ± 16.9	0.578
**Total bilirubin (µmol/l)**	1.93 ± 0.61	0.71 ± 0.18	**<0.001***	2.00 ± 0.93	0.73 ± 0.15	**<0.001***
**UCB (µmol/l)**	30.7 ± 12.5	9.73 ± 3.15	**<0.001***	32.5 ± 18.8	10.07 ± 3.80	**<0.001***
**BMI (kg/m²)**	24.1 [19.2; 29.7]	25.2 [19.4; 30.2]	1.000	21.9 [17.8; 25.4]	23.0 [18.3; 35.8]	1.000
**FM (%)**	26.4 ± 7.55	30.3 ± 8.57	0.423	22.2 ± 6.75	21.9 ± 8.34	1.000
**FFM (%)**	73.6 ± 7.55	68.1 ± 12.1	0.235	77.9 ± 6.76	78.1 ± 8.34	1.000
**WC (cm)**	86.6 ± 13.8	87.7 ± 11.2	0.990	77.2 ± 7.19	80.1 ± 13.0	0.847
**HC (cm)**	105 ± 8.80	106 ± 6.73	0.937	99.8 ± 5.53	101 ± 9.50	0.987
**WHR**	0.82 ± 0.08	0.83 ± 0.09	0.999	0.77 ± 0.04	0.79 ± 0.06	0.755
**Systolic BP (mmHg)**	117 ± 15.0	114 ± 11.8	0.906	112 ± 8.46	113 ± 11.9	0.989
**Diastolic BP (mmHg)**	77.7 ± 11.2	76.1 ± 10.3	0.969	70.3 ± 6.53	72.8 ± 11.2	0.839
**Glucose (mg/dl)**	82.5 ± 13.1	81.8 ± 6.49	0.995	79.9 ± 8.14	79.7 ± 8.50	1.000
**Triglycerides (mg/dl)**	69 [34; 359]	82 [50; 129]	0.501	55 [35; 113]	63 [33; 217]	0.657
**Total cholesterol (mg/dl)**	171 ± 35.4	187 ± 25.1	0.320	152 ± 25.4	159 ± 24.8	0.832
**LDL-C (mg/dl)**	92.5 ± 23.0	107 ± 18.6	0.091	81.4 ± 16.1	86.3 ± 18.0	0.833
**HDL-C (mg/dl)**	62.7 ± 14.3	57.7 ± 10.3	0.557	60.0 ± 11.8	57.6 ± 10.4	0.908
**IPAQ**	1.79 ± 0.79	1.42 ± 0.61	0.463	1.95 ± 0.86	1.90 ± 0.77	0.997
**VO2max pred**	39.3 ± 10.9	32.4 ± 9.7	0.091	40.3 ± 6.77	40.3 ± 8.06	1.000
**REE (kcal/ kg BW)**	23.5 ± 5.09	21.8 ± 5.94	0.726	25.8 ± 3.88	25.6 ± 4.52	0.999
**RQ**	0.76 ± 0.06	0.79 ± 0.08	0.438	0.78 ± 0.05	0.78 ± 0.05	1.000

*Supplementary table 1: Baseline metabolic parameters of the GS and the C group, stratified by age. Values are displayed in mean (standard deviation) if the data was normally distributed and in median [min; max] if not. An ANOVA was applied when normal distribution was true, and otherwise, a Kruskal-Wallis-Test was performed. Ł VO2max was calculated according to a formula by Nunes et al (38). Significance was marked in bold letters with a *.*

Domain	GS	C	p-value
Physical health	61.2	60.9	0.870
Psychological health	71.4	68.6	0.144
Social relationship	77.9	77.9	0.985
Environment	88.2	86.1	0.266
Question 1	85.5	86.5	0.754
Question 2	80.3	77.7	0.506

Supplementary table 2 WHOQOL-Bref: comparison between GS and C per domain of the WHOQOL. No significant differences were observed between GS and C in any of the domains. P-values origine from a Mann-Whitney-U-test.

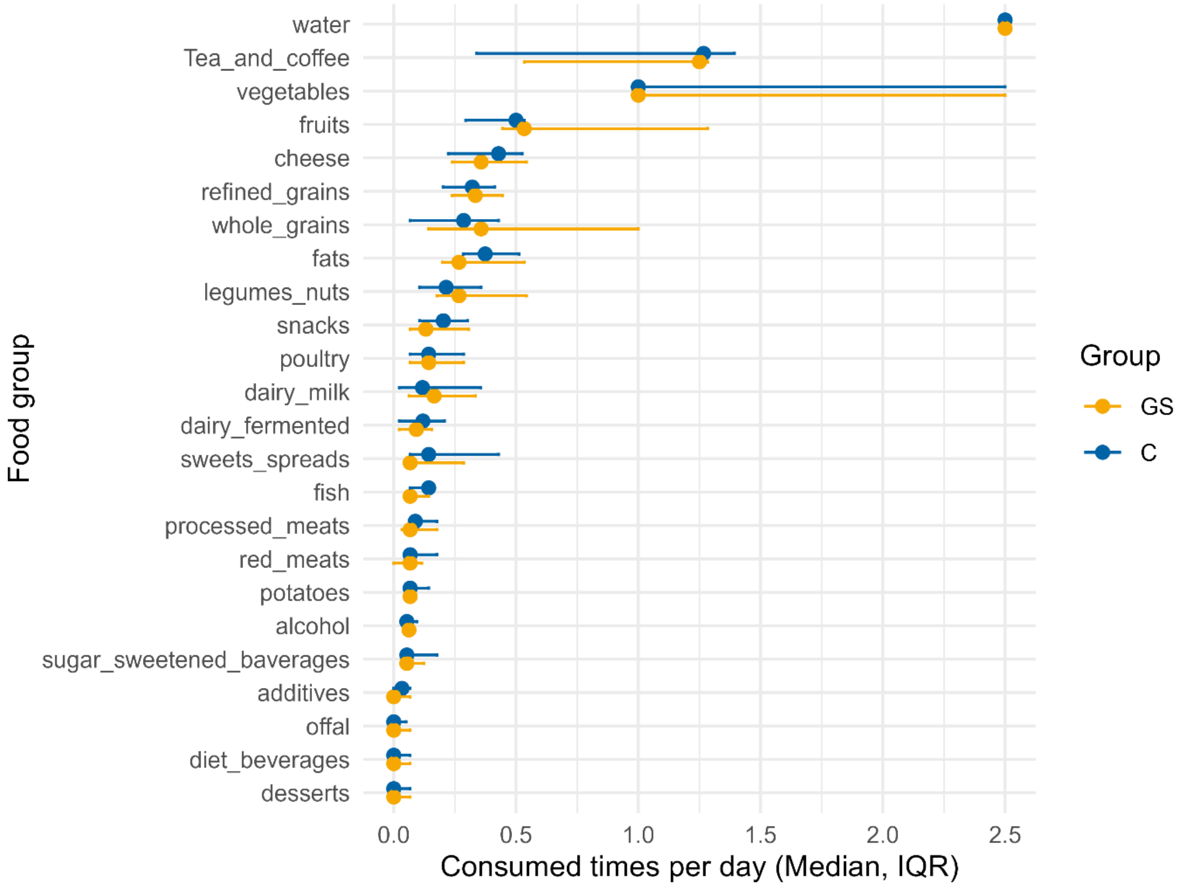

Supplementary figure 1 shows the results of the food frequency questionnaire (FFQ) per food group and calculated in consumed times per day for the GS (in yellow) and the C (in blue) group in Median ± Interquartile range (IQR). The food group refined grains contains white bread, mixed grain breads, pasta, cornflakes, and cereal dishes. Processed meats are sausages and hamburger patties. Fats are oil, butter, margarine, and other fats. Snacks are cakes, cookies, chocolate, and chips.

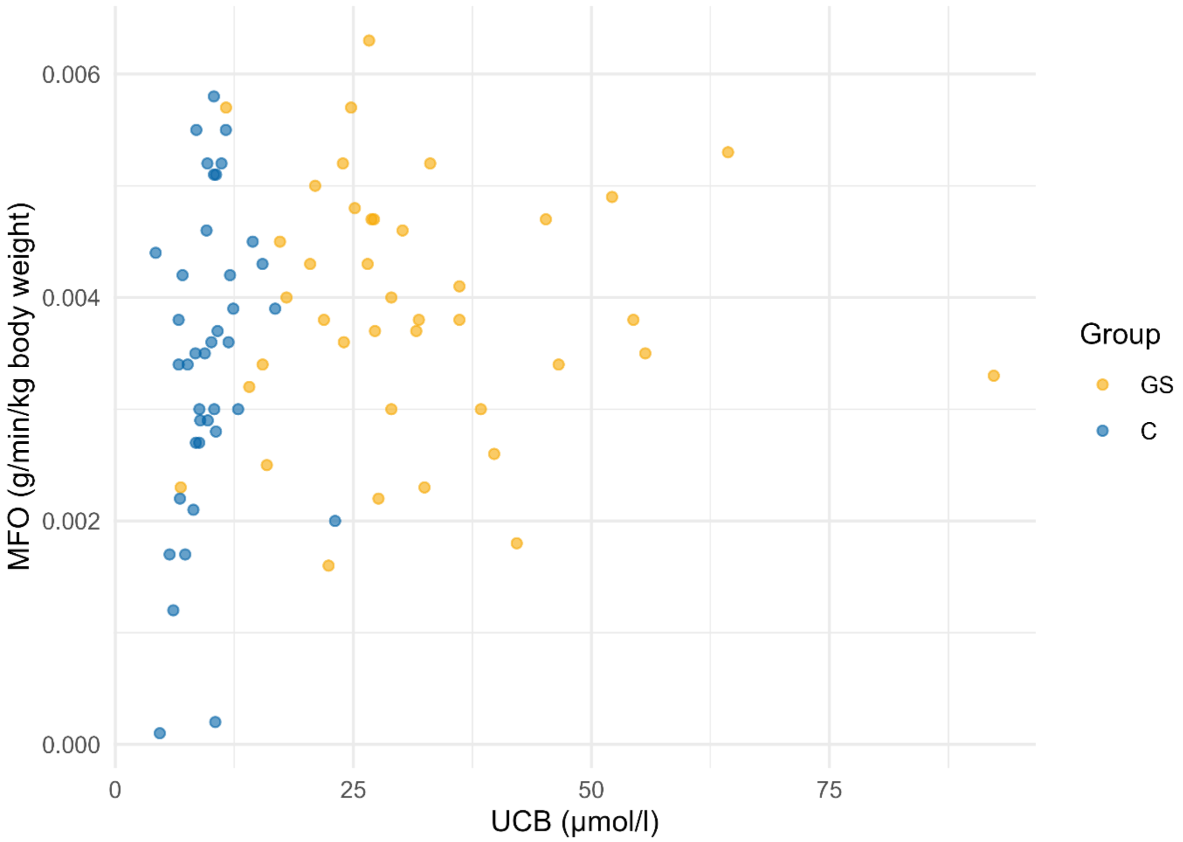

Supplementary figure 2 shows MFO (g/min/kg body weight) plotted against UCB (µmol/l).

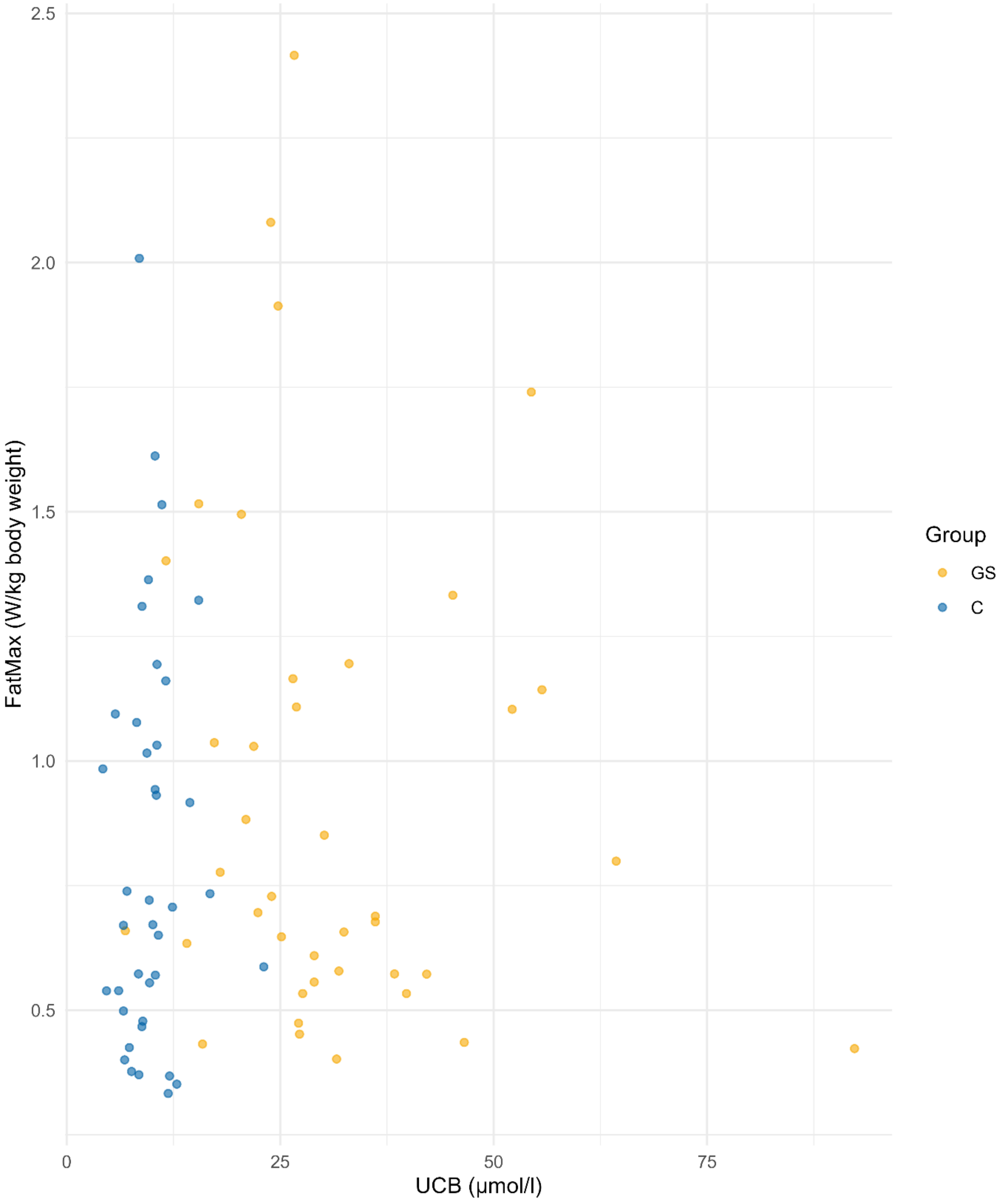

Supplementary figure 3 shows FatMax (W/kg body weight) plotted against UCB (µmol/l).

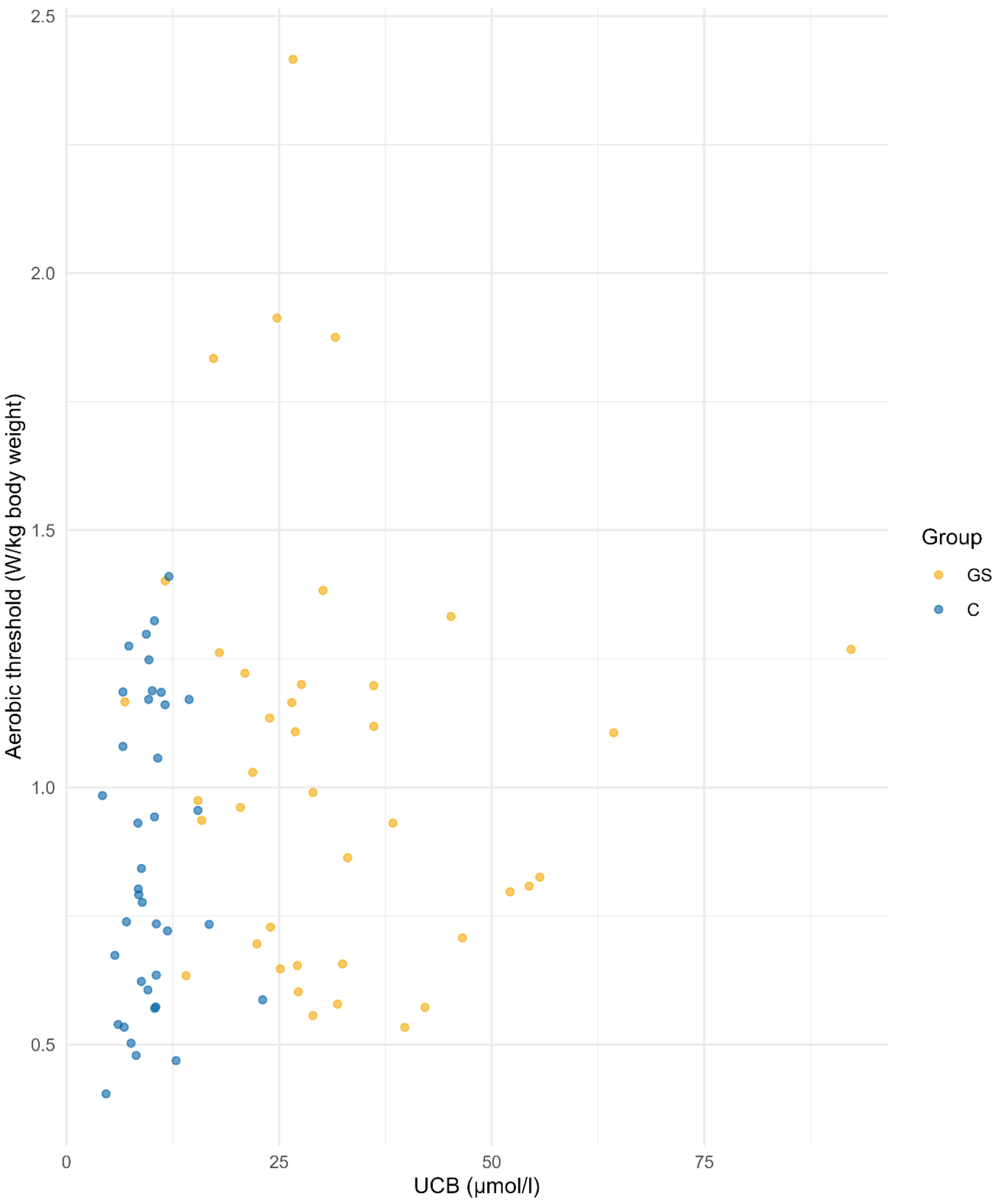

Supplementary figure 4 shows the aerobic threshold (W/kg body weight) plotted against UCB (µmol/l).

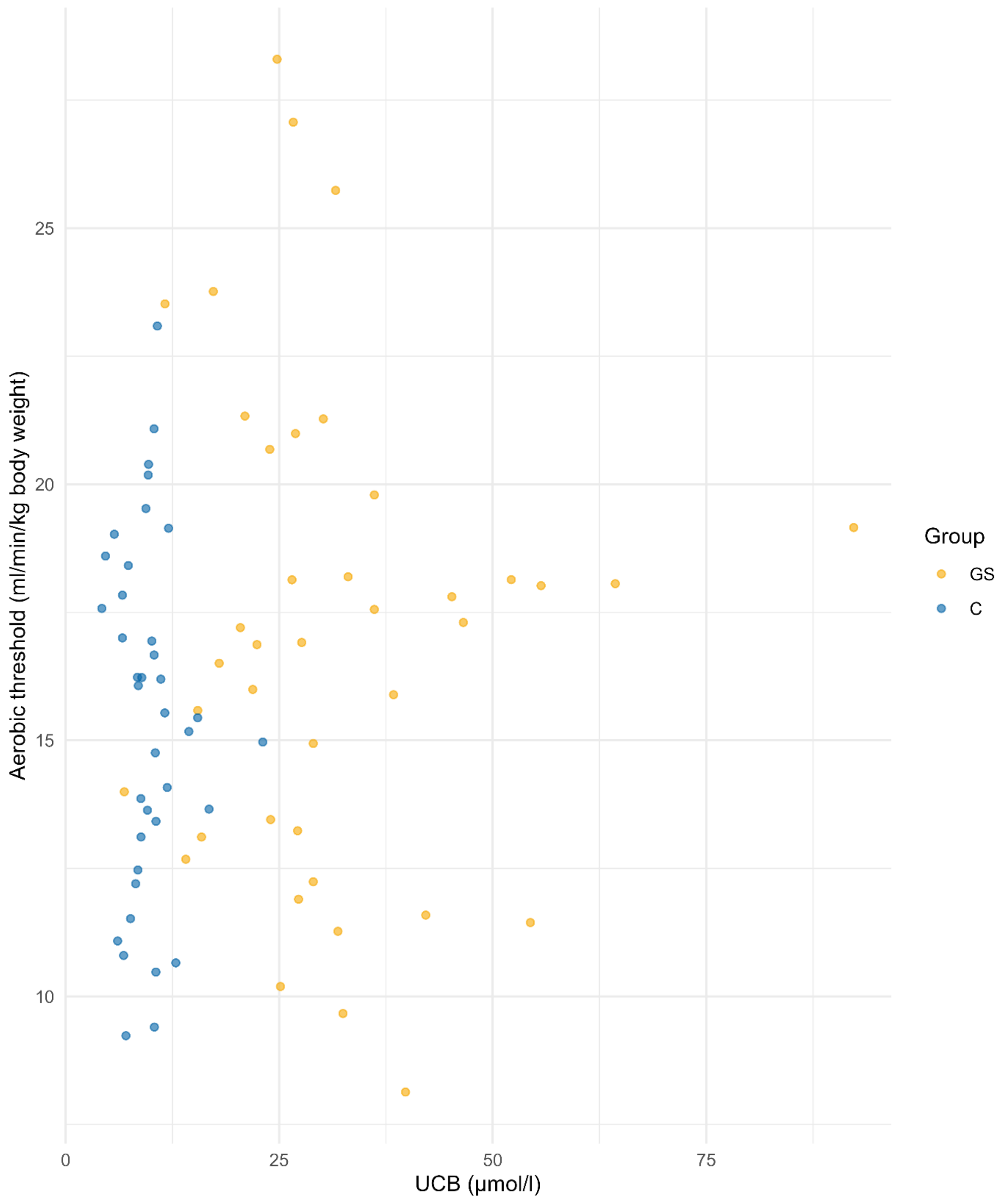

Supplementary figure 5 shows the aerobic threshold (ml/min/kg body weight) plotted against UCB (µmol/l).

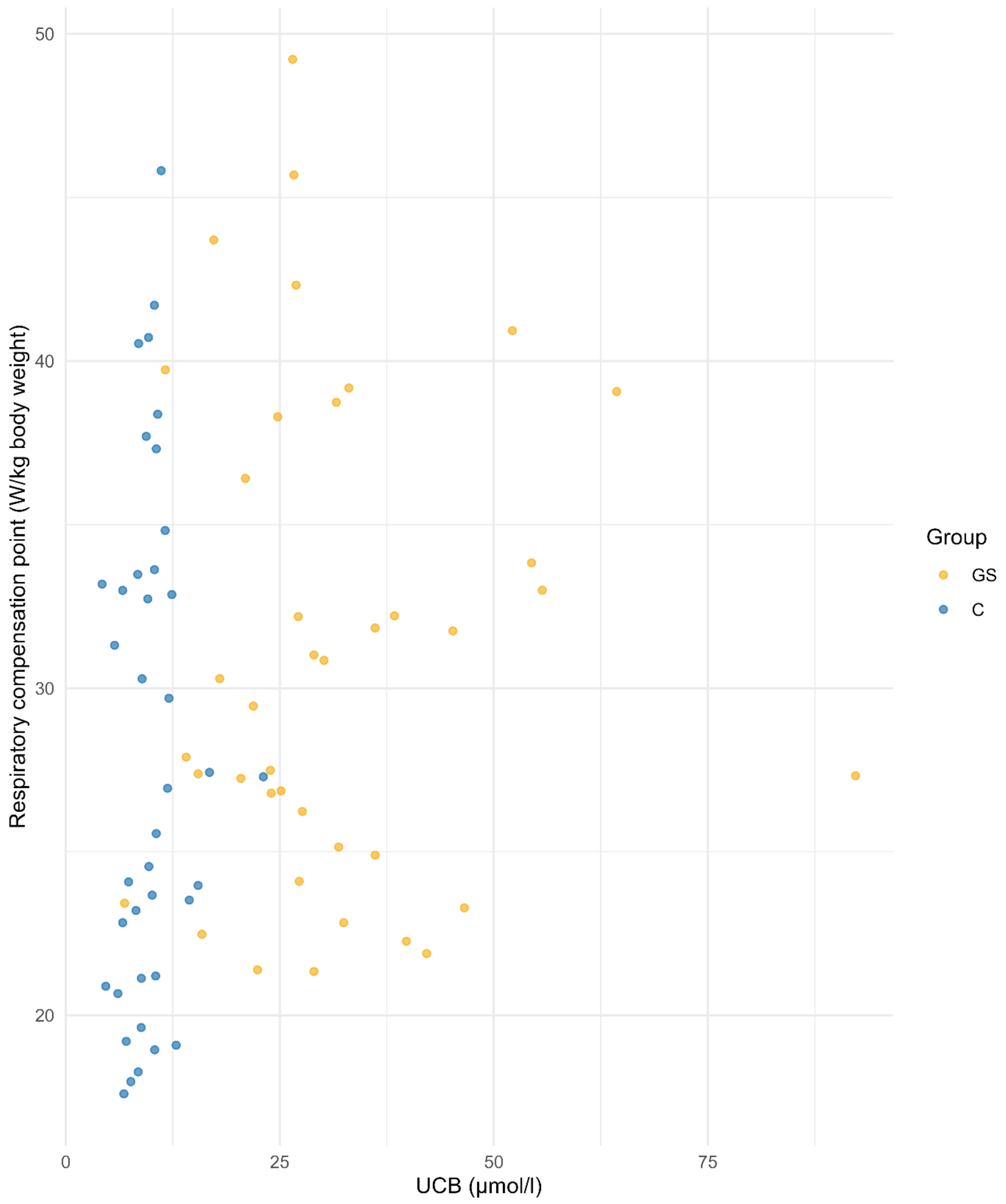

Supplementary Figure 6 shows the aerobic threshold (W/kg body weight) plotted against UCB (µmol/l).

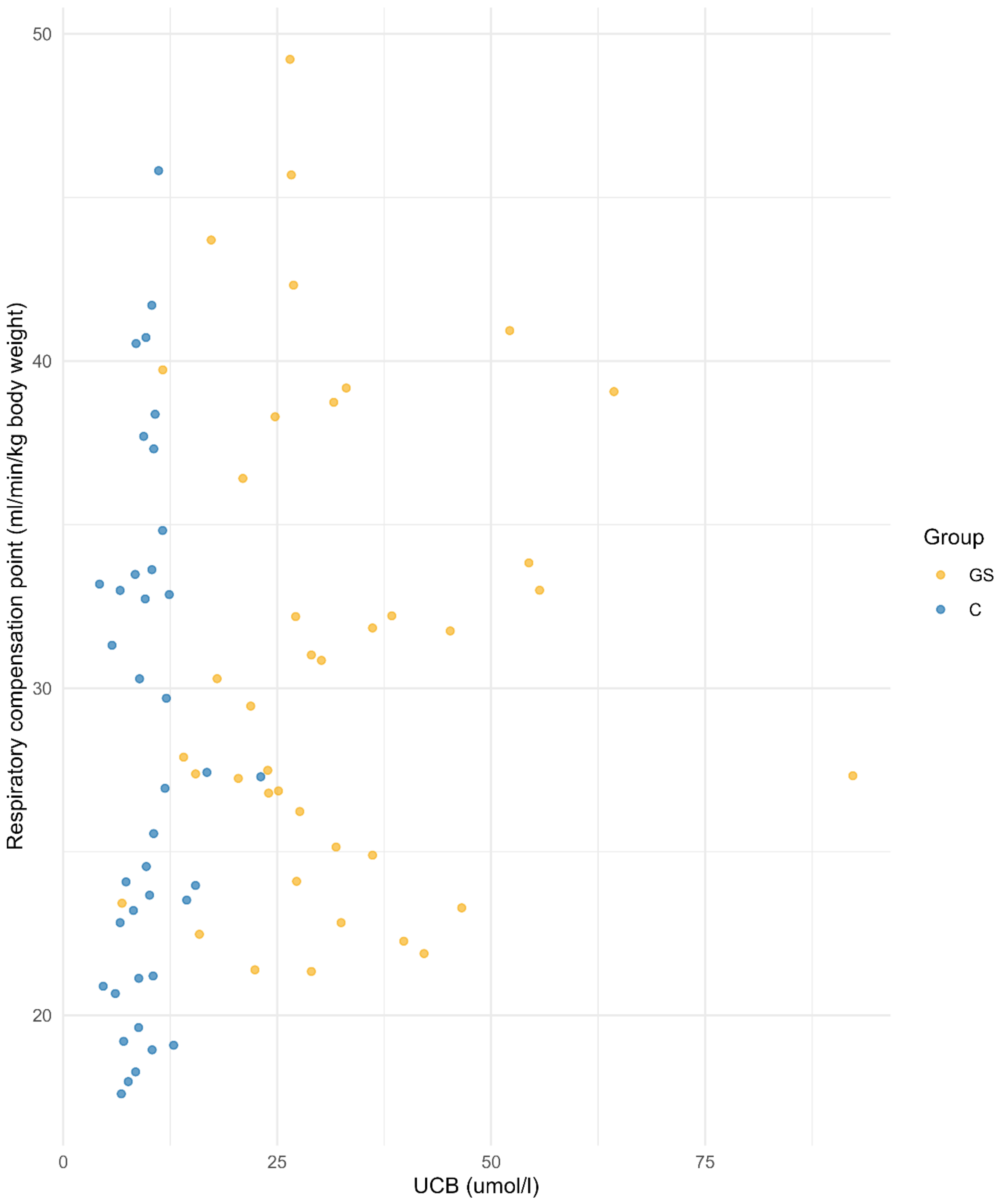

Supplementary figure 7 shows the respiratory compensation point (ml/min/kg body weight) plotted against UCB (µmol/l).
